# Tackling antimicrobial resistance in the COVID-19 pandemic

**DOI:** 10.2471/BLT.20.268573

**Published:** 2020-07-01

**Authors:** Haileyesus Getahun, Ingrid Smith, Kavita Trivedi, Sarah Paulin, Hanan H Balkhy

**Affiliations:** a Department of Global Coordination and Partnership on Antimicrobial Resistance, World Health Organization, avenue Appia 20, 1211 Geneva 27, Switzerland.; b Antimicrobial Resistance Division, World Health Organization, Geneva, Switzerland.

Antimicrobials have enabled medical advancements over several decades. However, the continuous emergence of resistance to antimicrobials restricts our ability to treat diseases and curbs efforts to achieve universal health coverage and the health-related sustainable development goal. Antimicrobial resistance is a neglected global crisis that requires urgent attention and action.^1^

Appropriate prescription and optimized use of antimicrobials guide the principles of antimicrobial stewardship activities, together with quality diagnosis and treatment, and reduction and prevention of infections.^2^ During the current coronavirus disease 2019 (COVID-19) pandemic there are potential threats that could affect antimicrobial stewardship activities and drive antimicrobial resistance. For instance, many individuals presenting with mild disease without pneumonia or moderate disease with pneumonia receive antibiotics.^3^ A review of studies published on hospitalized COVID-19 patients identified that while 72% (1450/2010) of patients received antibiotics, only 8% (62/806) demonstrated superimposed bacterial or fungal co-infections.^4^ WHO also reports that azithromycin is being widely used with hydroxychloroquine although it is not yet recommended outside of COVID-19 clinical trials.^3^

Furthermore, hospital admissions increase the risk of health-care-associated infections and the transmission of multidrug-resistant organisms, which in turn lead to increased antimicrobial use.^5^ A recent study conducted in intensive care units in 88 countries showed that although only 54% (8135/15 165) of patients had suspected or proven bacterial infection, 70% (10 640/15 165) of them had received at least one antibiotic either for prophylaxis or treatment purposes.^6^

Disruptions to health services during the pandemic are causing interruptions to treatments, such as for tuberculosis and human immunodeficiency virus, which can also lead to selection for drug resistance. Similarly, disruption to vaccination services can increase risk of infection, potentially leading to an overuse of antimicrobials.

Another potential threat is the wide use of biocidal agents for environmental and personal disinfection, including in non-health-care settings. Low level exposure to biocidal agents can select for drug-resistant strains and enhance the risk of cross resistance to antibiotics,^7^ particularly those that treat Gram-negative bacteria.^8^

The latest update of WHO’s interim guidance on the clinical management of COVID-19 incorporates antibiotic stewardship principles with specific recommendations.^3^ The guidance does not recommend antibiotic therapy or prophylaxis for patients with mild or moderate COVID-19 unless signs and symptoms of a bacterial infection exist. The use of empiric antibiotic treatment for patients with suspected or confirmed severe COVID-19, based on clinical judgement considering patient host factors and local epidemiology, along with daily assessments for de-escalation, is recommended.^3^ The guidance further states that empiric antibiotic bacterial pneumonia treatment can be considered in older people residing in long-term care facilities and in children younger than five years with moderate COVID-19. As these are non-hospitalized patients, antibiotics within WHO’s AWaRe (access, watch, reserve) classification of antibiotics categorized as access, such as co-amoxicillin, should preferably be administered.^9^

We argue that antimicrobial stewardship activities should be integrated into the pandemic response across the broader health system through five measures. 

First, increase clinical competence among health workers treating COVID-19 patients through targeted training. Key competencies include ability to identify signs and symptoms of severe COVID-19 and that of a superimposed bacterial or fungal disease; eliminate unnecessary antibiotic use including daily de-escalation; evaluate the need for medical devices and others that increase the chances of health-care-associated infections and antibiotic use; and implement strict infection prevention and control measures. Second, ensure the continuity of essential health services and regular supply of quality assured and affordable antimicrobials including antiretroviral and tuberculosis drugs, and vaccines. Third, reduce the turnaround time of COVID-19 testing by improving testing methods and expanding testing facilities, especially for presumed patients, to reduce the urge to initiate antibiotics. Fourth, exercise maximum caution in the use of biocides for environmental and personal disinfection and prioritize biocidal agents without or with a low selection pressure for antibiotic resistance. Fifth, address gaps in research to ensure that antimicrobial stewardship activities become an integral part of the pandemic response and beyond. The research agenda includes: rapid and affordable diagnostic tests that differentiate between bacterial and viral respiratory tract infections; the short- and long-term impact of wide use of biocides for environmental and personal disinfection including cross resistance to antimicrobials; and potential alternatives for sustainable environmental and personal disinfection.

These measures would stem the emergence of untreatable drug-resistant infections and diseases that could potentially lead to another public health emergency.

## References

[1] No time to wait: securing the future from drug resistant infections. IACG Report to the Secretary General of the United Nations, April 2019. Geneva: The UN Interagency Coordination Group on AMR; 2019. Available from: https://www.who.int/antimicrobial-resistance/interagency-coordination-group/IACG_final_report_EN.pdf [cited 2020 Jun 1].

[2] Antimicrobial stewardship programmes in health-care facilities in low- and middle-income countries: a WHO practical toolkit. Geneva: World Health Organization; 2019. Available from: https://apps.who.int/iris/handle/10665/329404 [cited 2020 Jun 4]. 10.1093/jacamr/dlz072 PMC8210188 34222945

[3] Clinical management of COVID-19 Interim Guidance - May 2020. Geneva: World Health Organization; 2020. Available from: https://www.who.int/publications-detail/clinical-management-of-covid-19 [cited 2020 Jun 4].

[4] Rawson TM, Moore LSP, Zhu N, Ranganathan N, Skolimowska K, Gilchrist M, et al. Bacterial and fungal co-infection in individuals with coronavirus: A rapid review to support COVID-19 antimicrobial prescribing. Clin Infect Dis. 2020 5 2;ciaa530. 10.1093/cid/ciaa530 32358954 PMC7197596

[5] Saleem Z, Godman B, Hassali MA, Hashmi FK, Azhar F, Rehman IU. Point prevalence surveys of health-care-associated infections: a systematic review. Pathog Glob Health. 2019 6;113(4):191–205. 10.1080/20477724.2019.1632070 31215326 PMC6758614

[6] Vincent JL, Sakr Y, Singer M, Martin-Loeches I, Machado FR, Marshall JC, et al.; EPIC III Investigators. Prevalence and outcomes of infection among patients in intensive care units in 2017. JAMA. 2020 3 24;323(15):1478–87. 10.1001/jama.2020.2717 32207816 PMC7093816

[7] Caselli E. Hygiene: microbial strategies to reduce pathogens and drug resistance in clinical settings. Microb Biotechnol. 2017 9;10(5):1079–83. 10.1111/1751-7915.12755 28677216 PMC5609343

[8] Kampf G. Biocidal agents used for disinfection can enhance antibiotic resistance in gram-negative species. Antibiotics (Basel). 2018 12 14;7(4):110. 10.3390/antibiotics7040110 PMC6316403 30558235

[9] The AWaRe classification of antibiotics database. Geneva: World Health Organization; 2019. Available from: https://adoptaware.org/ [cited 2020 Jun 4].

